# Inflammatory Cytokine Variations After Non-Surgical Periodontal Therapy Across Periodontal Stages and Grades

**DOI:** 10.3390/dj13120591

**Published:** 2025-12-09

**Authors:** Mirlinda Sopi Krasniqi, Zana Sllamniku Dalipi, Donika Bajrami Shabani, Etleva Droboniku, Gramos Begolli, Gerta Kaçani, Aida Meto

**Affiliations:** 1 Department of Periodontology and Oral Medicine, University Dentistry Clinical Center of Kosovo, Medical Faculty, University of Pristina, 10000 Pristina, Kosovo; mirlinda.sopi@uni-pr.edu; 2 Department of Pathology and Endodontics, University Dentistry Clinical Center of Kosovo, Medical Faculty, University of Pristina, 10000 Pristina, Kosovo; donika.bajrami@uni-pr.edu; 3 Department of Dental Therapy, Faculty of Dental Medicine, University of Medicine, 1005 Tirana, Albania; etleva.droboniku@umed.edu.al; 4 Department of Biochemistry, University Clinical Center of Kosovo, 10000 Prishtina, Kosovo; gramos.begolli@kolegji-heimerer.eu; 5 Department of Prosthodontics, Faculty of Dental Medicine, University of Medicine, 1005 Tirana, Albania; gerta.kacani@umed.edu.al; 6 Department of Dentistry, Faculty of Dental Sciences, University of Aldent, 1007 Tirana, Albania; 7 Department of Surgery, Medicine, Dentistry and Morphological Sciences with Interest in Transplant, Oncology and Regenerative Medicine, University of Modena and Reggio Emilia, 41125 Modena, Italy; 8 Department of Dental Research Cell, Dr. D. Y. Patil Dental College and Hospital, Dr. D.Y. Patil Vidyapeeth, Pimpri, Pune 411018, India

**Keywords:** cytokines, C-reactive protein, periodontitis, periodontal therapy, systemic inflammation

## Abstract

**Background:** Periodontitis is associated with systemic inflammation; however, the relationship between disease severity and systemic inflammatory biomarkers remains unclear. This study aimed to evaluate the association between periodontitis stage and grade with systemic levels of C-reactive protein (CRP), interleukin-1β (IL-1β), interleukin-6 (IL-6), and tumor necrosis factor-alpha (TNF-α) and assess changes following standardized non-surgical periodontal therapy. **Methods:** Patient records from the University Dentistry Clinical Center of Kosovo were reviewed. Periodontitis was classified using the 2018 staging and grading system. Periodontal parameters (probing pocket depth, clinical attachment loss, bleeding on probing, plaque index, and gingival index) were assessed at six sites per tooth (excluding third molars). Serum levels of IL-1β, IL-6, TNF-α, and high-sensitivity CRP were measured before and after therapy using high-sensitivity ELISA. Blood samples were centrifuged, and serum was stored at −20 °C. All patients underwent standardized non-surgical periodontal therapy, including full-mouth scaling and root planning, without systemic antibiotics. Data were analyzed using SPSS v22.0. **Results:** Among the patients, 28.0% had Stage I–II, 40.0% Stage III, and 32.0% Stage IV periodontitis; 29.3% were Grade A, 45.3% Grade B, and 25.3% Grade C. At baseline, all systemic inflammatory biomarkers (CRP, IL-1β, IL-6, and TNF-α) were significantly higher in periodontitis patients compared with the control group, indicating an increased systemic inflammatory burden before therapy. After therapy, significant reductions in CRP, IL-1β, IL-6, and TNF-α were observed across all stages and grades (all *p* < 0.01), indicating a decrease in systemic inflammatory burden. **Conclusions:** Non-surgical periodontal therapy significantly lowers systemic inflammatory biomarkers regardless of periodontitis severity, supporting their role as indicators of disease activity and treatment response.

## 1. Introduction

Periodontitis is a chronic multifactorial inflammatory disease characterized by progressive destruction of the tooth-supporting structures, primarily driven by dysbiotic dental biofilms and an exacerbated host immune response. In 2017, the World Workshop on the Classification of Periodontal and Peri-Implant Diseases and Conditions introduced a refined framework for diagnosing and categorizing periodontitis based on staging and grading [1]. Diagnosis requires clinical attachment loss (CAL) at two or more non-adjacent teeth or buccal/oral CAL ≥ 3 mm with pocketing >3 mm at two or more teeth, excluding attachment loss due to non-periodontal causes. Staging reflects the severity and complexity of management, ranging from initial to advanced stages, while grading estimates the rate of progression and accounts for risk factors such as smoking and diabetes. Alterations in immune regulation, accompanied by elevated circulating concentrations of pro-inflammatory mediators including IL-1β, IL-6, IL-1, tumor necrosis factor-alpha (TNF-α), and C-reactive protein (CRP), can be observed even during the initial stages of disease development [2].

**Figure 1:**
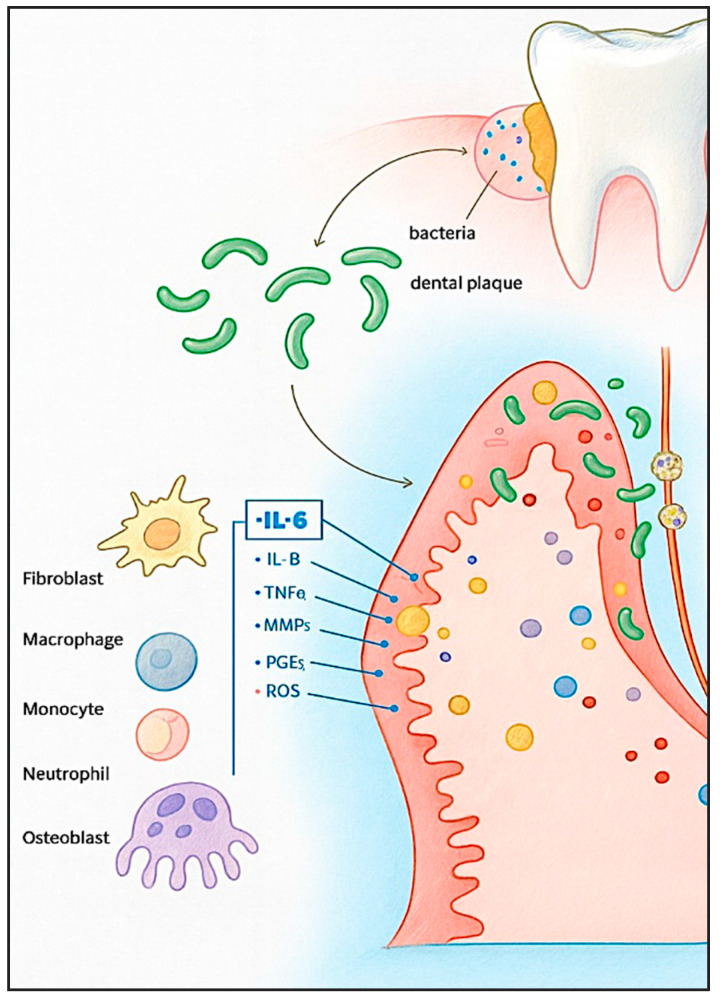
Inflammatory mechanisms in periodontitis. Periodontal pathogens interact with host cells, initiating alterations in cellular activity and promoting the secretion of inflammatory mediators.

In response to bacterial biofilm, immune cells secrete pro-inflammatory cytokines IL-1β and TNF-α. These cytokines trigger the production of downstream mediators, including interleukin-6 (IL-6), matrix metalloproteases (MMPs), and prostaglandin E_2_ (PGE_2_) (Figure 1). Together, these factors contribute to the degradation of connective tissue and the activation of osteoclasts, ultimately resulting in alveolar bone resorption [3].

Elevated gingival crevicular fluid (GCF) levels of IL-6, IL-17, and IL-35 have been reported in patients with Stage III–IV periodontitis compared with healthy controls, with IL-17 showing a strong correlation with probing depth [4]. Similarly, increased salivary concentrations of IL-1β and RANKL have been observed in individuals with Stage III/IV and Grade C disease, with these markers associated with both greater disease severity and accelerated progression [5]. Salivary IL-17 and IL-10 have been shown to differentiate healthy individuals from those with periodontitis and to discriminate among disease stages [6]. Salivary IL-1β levels increase progressively from gingivitis to Stage II–III periodontitis, indicating its diagnostic relevance in periodontal disease progression [7]. Increased concentrations of IL-1β, TNF-α, and VEGF have also been observed in Grade C sites of Stage III periodontitis, reinforcing the link between cytokine expression and disease aggressiveness [8].

Non-surgical periodontal therapy (NSPT), primarily through scaling and root planing, remains the cornerstone treatment and has been shown to reduce local and systemic inflammation. Significant reductions in gingival crevicular fluid (GCF) TNF-α and IL-10 levels have been observed following non-surgical periodontal therapy (NSPT) in Stage III Grade B periodontitis [9]. Systematic reviews and meta-analyses consistently report decreases in IL-6 and other inflammatory mediators after therapy across populations with different systemic conditions, including obesity [10], diabetes [11], and hypertension [12]. NSPT significantly reduced salivary inflammatory cytokines (IL-1β, TNF-α, MMP-8), although the magnitude of improvement was influenced by systemic factors [13].

Despite these advances, limited studies have simultaneously evaluated systemic inflammatory biomarkers across different periodontal stages and grades within the same cohort, both before and after NSPT, while controlling for confounders such as age, sex, smoking, and systemic diseases. This study aims to address these gaps by correlating serum CRP, IL-1β, IL-6, and TNF-α levels with periodontal stage and grade according to the 2018 classification, and by assessing their changes following NSPT. We aim to assess the association between periodontal disease stage and grade and systemic inflammatory biomarkers, C-reactive protein (CRP), interleukin-1 beta (IL-1β), interleukin-6 (IL-6), and tumor necrosis factor-alpha (TNF-α) in patients with periodontitis and evaluate the changes in these biomarkers following standardized non-surgical periodontal therapy.

## 2. Materials and Methods

### 2.1. Study Design and Ethical Approval

This retrospective observational study utilized pre-existing clinical and biochemical records of patients who had previously undergone standardized non-surgical periodontal therapy at the University Dental Clinical Center of Kosovo. No additional biological samples were collected, and no prospective interventions were undertaken for this investigation. The analysis included patient data obtained from the Department of Periodontology and Oral Medicine, covering the period from January 2011 to December 2012. The study protocol was reviewed and approved by the Joint Ethics Committee of the University Dentistry Clinical Center of Kosovo (Approval No. 1551; date: 8 October 2010). Written informed consent was obtained from all participants in accordance with the ethical principles outlined in the Declaration of Helsinki.

### 2.2. Classification and Study Variables

Periodontal diagnosis, staging, and grading were retrospectively determined based on the criteria established by the 2018 World Workshop on the Classification of Periodontal and Peri-Implant Diseases and Conditions [1]. Clinical periodontal parameters were compared with serum levels of inflammatory biomarkers, including C-reactive protein (CRP), interleukin-1 beta (IL-1β), interleukin-6 (IL-6), and tumor necrosis factor-alpha (TNF-α), before and after non-surgical periodontal therapy.

### 2.3. Participants

**Figure 2:**
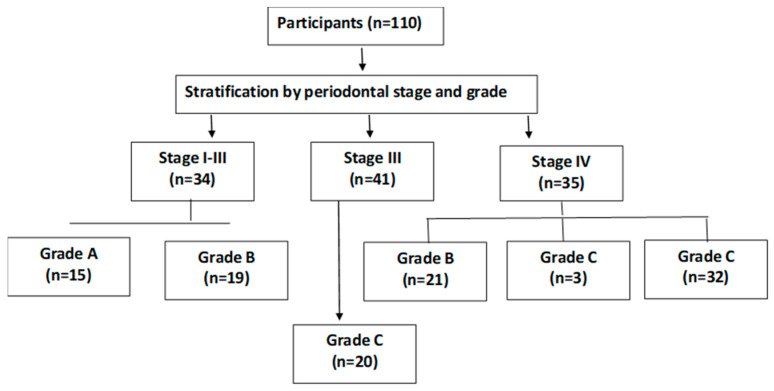
Patient distribution by periodontal stage and grade.

A total of 110 participants were included and categorized into three groups based on periodontal status:

- Group I: Periodontally healthy individuals or those with gingivitis (n = 35);
- Group II: Patients with Stage I–II periodontitis (n = 35);
- Group III: Patients with advanced Stage III–IV periodontitis (n = 40) (Figure 2).

Inclusion criteria comprised adults aged ≥18 years with complete clinical and radiographic records. Only systemically healthy, non-diabetic individuals were included based on the available medical records. Smoking status was recorded qualitatively as smoker or non-smoker.

Exclusion criteria included a history of periodontal treatment within the preceding six months, use of systemic antibiotics within the last three months, presence of acute infection at the time of examination, pregnancy or lactation, systemic diseases, or medications known to influence systemic inflammation (e.g., corticosteroids or immunosuppressants).

### 2.4. Clinical Examination

Comprehensive periodontal assessments were performed at six sites per tooth, excluding third molars. The following parameters were recorded:

- Probing Pocket Depth (PPD);
- Clinical Attachment Level (CAL);
- Bleeding on Probing (BOP);
- Plaque Index (PI) according to Silness and Löe [14];
- Gingival Index (GI) according to Löe and Silness [15].

### 2.5. Periodontal Treatment

Patients diagnosed with periodontitis received standardized non-surgical periodontal therapy, including full-mouth scaling and root planning under local anesthesia, complemented by individualized oral hygiene instructions. No systemic antibiotics were prescribed during the study period. Clinical and biochemical parameters were reassessed following therapy.

### 2.6. Biochemical Analysis

All participants provided written informed consent before data collection, allowing the use of their anonymized clinical and biochemical data for research purposes. Venous blood samples (10 mL) were collected from each participant using sterile venipuncture into tubes without anticoagulants. Samples were centrifuged at 2000 rpm for 10 min to obtain serum, which was aliquoted into sterile 1.7 mL Eppendorf tubes and stored at −20 °C until analysis. Serum levels of IL-1β, IL-6, TNF-α, and high-sensitivity CRP (hs-CRP) were quantified using high-sensitivity ELISA kits (IBL International GmbH, Hamburg, Germany), following the manufacturer’s instructions.

### 2.7. Statistical Analysis

As this study employed a retrospective design, the sample size encompassed all eligible cases within the specified timeframe. A post hoc power analysis, informed by effect sizes reported in comparable cytokine research, demonstrated that the available sample provided sufficient statistical power (>80%) to detect moderate differences between groups at a 5% significance threshold.

Data analysis was conducted using IBM SPSS Statistics for Windows, Version 22.0 (IBM Corp., Armonk, NY, USA). Descriptive statistics, including means, standard deviations, and ranges, were computed. For parametric data, the *t*-test and one-way ANOVA with Bonferroni post hoc adjustment were used, whereas the Chi-square (χ^2^) and Wilcoxon matched-pairs signed-rank tests were used for nonparametric data. Statistical significance was defined as *p* < 0.05. Normality of data was evaluated using the Shapiro–Wilk test. Parametric or non-parametric tests were applied accordingly. For post-hoc pairwise comparisons, Bonferroni correction was used to adjust for multiple testing, and an adjusted *p*-value < 0.05 was considered statistically significant. The correlation between clinical periodontal parameters and biochemical parameters was assessed using the Spearman correlation.

## 3. Results

No statistically significant differences were found between the groups regarding gender, place of residence, or educational attainment (*p* > 0.05). However, the groups differed significantly in mean age (*p* < 0.0001). Patients with periodontitis were staged as follows: 21 (28.0%) in stage I–II, 30 (40.0%) in stage III, and 24 (32.0%) in stage IV. Regarding grading, 22 (29.3%) were grade A, 34 (45.3%) grade B, and 19 (25.3%) grade C (Table 1).

CRP values were significantly lower in the control group than in all stages and grades, both at baseline and post-treatment (*p* < 0.05). Within-group comparisons (Wilcoxon matched-pairs signed-rank test) showed significant reductions from baseline to post-treatment for stage I–II (*p* = 0.0010), stage III (*p* = 0.0005), and stage IV (*p* < 0.0001). By grade, significant decreases were observed for grade A (*p* = 0.0020) and grade C (*p* < 0.0001) using Wilcoxon; for grade B, the change was significant using the independent *t*-test (*p* = 0.0012) (Table 2).

IL-1 levels were consistently lower in the control group compared with all stages and grades of periodontitis at both assessed time points (*p* < 0.05). Following non-surgical periodontal treatment, significant within-group reductions from baseline were observed across all stages: stage I–II (*p* = 0.0001), stage III (*p* < 0.0001), and stage IV (*p* = 0.0001). Similarly, analyses by grade demonstrated notable decreases for grade A (*p* < 0.0001), grade B (*p* < 0.0001), and grade C (*p* = 0.0005), as determined using the Wilcoxon signed-rank test (Table 3), indicating a consistent treatment-related decline in IL-1 levels irrespective of disease severity.

IL-6 levels were significantly lower in the control group compared with all stages and grades of periodontitis at both assessment points (*p* < 0.05). Within-group analyses demonstrated significant reductions from baseline to post-treatment for stage I–II (*p* = 0.0002), stage III (*p* < 0.0001), and stage IV (*p* = 0.0002), as evaluated by the Wilcoxon signed-rank test. Similarly, stratification by grade revealed significant decreases for grade A (*p* = 0.0001), grade B (*p* < 0.0001), and grade C (*p* = 0.001) (Table 4), indicating a consistent treatment-related decline in IL-6 levels across all levels of disease severity.

At baseline, TNF-α levels were significantly lower in the control group compared with all stages and grades of periodontitis (*p* < 0.05). Within-group analyses revealed significant reductions from baseline to post-treatment for stage I–II (*p* = 0.0001), stage III (*p* = 0.0001), and stage IV (*p* < 0.0001), as assessed by the Wilcoxon signed-rank test. Stratification by grade demonstrated comparable decreases, with significant reductions observed for grade A (*p* < 0.0001), grade B (*p* < 0.0001), and grade C (*p* = 0.0005) (Table 5).

Although the levels of CRP, IL-1, and TNF were higher in stage IV compared to other stages, no statistically significant differences were observed (*p* > 0.05). A significant difference was found only in IL-6 values according to stages at the beginning of treatment (*p* = 0.025) and at the end of treatment (*p* = 0.009) (Table 6).

Although the levels of CRP, IL-1, IL-6, and TNF were higher in grade C compared to the other grades, no statistically significant difference was observed (*p* > 0.05) (Table 7).

When combining stages and grades, a statistically significant difference was observed only in the levels of IL-6 (*p* < 0.05) (Table 8).

On the other hand, a significant negative correlation was observed between the level of epithelial adhesion and systemic inflammatory biomarkers, including CRP, IL-1, IL-6, and TNF-α (Table 9). This suggests that improvements in local periodontal healing, reflected by improved epithelial adhesion, are associated with decreased systemic inflammatory activity. Although no statistically significant correlations were found between other clinical parameters (BOP, PPD, CAL) and cytokine levels, the observed association with epithelial adhesion supports the hypothesis that effective periodontal therapy contributes to both local tissue repair and systemic inflammatory modulation.

## 4. Discussion

Compared to a previous study of 136 chronic periodontitis patients classified by the 1999 criteria, our cohort showed notable differences in disease distribution. While the earlier study had most patients in Stage II (65%) and fewer in Stages III and IV (28%), our sample had a greater proportion of advanced cases, with Stages III and IV comprising 72%. Both studies reported a similar rate of Grade C patients (~25%), but our group had more Grade A (29.3% vs. 11%) and fewer Grade B patients (45.3% vs. 63%) [16].

CRP is part of the pentraxin protein family involved in the innate immune response and serves as a highly sensitive and dependable biomarker of systemic inflammation and overall inflammatory activity in the body [17]. Elevated CRP levels reflect persistent tissue damage and may serve as a marker of worsening local disease severity [18]. In our study, CRP levels significantly decreased after treatment across all stages (Stage I–II: *p* = 0.001; Stage III: *p* = 0.0005; Stage IV: *p* < 0.0001) using the Wilcoxon signed-rank test. Significant reductions were also noted across grades, with Grade A (*p* = 0.002) and Grade C (*p* < 0.0001) by the Wilcoxon test. A systematic review and meta-analysis by Machado et al. demonstrated that patients with aggressive periodontitis exhibited significantly higher CRP levels compared to those with chronic periodontitis [19].

The expression of IL-6, along with other pro-inflammatory cytokines such as IL-1, IL-2, IL-3, and TNF-α, can be upregulated in response to stimuli like bacteria, viruses, parasites, and lipopolysaccharides from microbial cell walls. IL-6 is a glycoprotein cytokine with a molecular weight ranging from 19 to 28 kDa, encoded by a gene located on the short arm of chromosome 7. It is produced by various cell types, including monocytes and lymphocytes [20]. The IL-1 gene family comprises 11 genes clustered within a 430-kb region on the long arm of chromosome 2 (2q12–q21). These genes encode IL-1α and IL-1β, which promote the expression of endothelial adhesion molecules and stimulate the release of additional cytokines, thereby enhancing neutrophil recruitment [21].

In our study, IL-1 and IL-6 levels showed statistically significant reductions following treatment across all periodontal stages (I–IV) and grades (A–C), with all comparisons yielding *p* < 0.001 based on the Wilcoxon matched-pairs signed-rank test. These findings are consistent with those reported by Kabacaoğlu et al. [22], who also observed significant decreases in pro-inflammatory cytokine levels following periodontal therapy. Similarly, Bertoldi et al. [23] demonstrated that salivary levels of MMP-8, IL-1β, IL-4, IL-8, and IL-10 significantly differ between healthy individuals, untreated periodontitis patients, and those post-treatment, highlighting the potential of these biomarkers for non-invasive diagnosis and monitoring of periodontal disease progression and treatment response.

In addition to other inflammatory mediators, TNF-α plays a pivotal role in periodontal tissue destruction. As one of the most potent pro-inflammatory cytokines involved in both joint and periodontal inflammation, TNF-α promotes osteoclast differentiation from monocyte/macrophage lineages, upregulates RANKL expression in osteoblasts, and drives bone resorption. Moreover, it amplifies disease progression by inducing MMPs production and enhancing inflammatory cell infiltration, ultimately contributing to extracellular matrix degradation and alveolar bone loss [24,25,26]. The findings of this study align with established evidence, as elevated TNF-α levels in periodontitis patients reinforce its critical role in periodontal inflammation, connective tissue degradation, and alveolar bone resorption. Following treatment, TNF-α levels significantly decreased across all periodontal stages, Stage I–II (*p* = 0.0001), Stage III (*p* = 0.0001), and Stage IV (*p* < 0.0001), as well as across all grades: Grade A (*p* < 0.0001), Grade B (*p* < 0.0001), and Grade C (*p* = 0.0005). These findings are in agreement with those who reported a statistically significant reduction in GCF and serum levels of leucine-rich α-2-glycoprotein (LRG), IL-6, and TNF-α following periodontal therapy, compared to baseline values (*p* < 0.001). Their results further support the reliability of these biomarkers as indicators of periodontal inflammatory activity [27].

These findings reinforce the pivotal role of inflammatory biomarkers in the pathogenesis and progression of periodontitis and demonstrate that periodontal therapy effectively reduces systemic inflammation across disease stages and grades. However, further research with larger, diverse cohorts and longer follow-up is needed to validate these biomarkers as reliable tools for diagnosis, prognosis, and treatment monitoring.

Quantitative data on smoking exposure, BMI, and other potential confounders were not consistently available in the retrospective dataset and could not be statistically adjusted. Although sensitivity analyses excluding smokers yielded similar outcomes, future prospective studies should incorporate detailed lifestyle and metabolic variables to more precisely evaluate their influence on systemic inflammatory responses.

In addition, psychosocial stress has been identified as an important modifier of periodontal inflammation and immune function. Macrì et al. [28] demonstrated that elevated stress levels were significantly associated with poorer periodontal health, underscoring the need to account for psychological variables in future analyses, while Villafuerte et al. [29] reported that psychological stress may reduce the effectiveness of periodontal therapy by sustaining systemic inflammatory activation. These findings underscore the need for future studies to consider psychological and behavioral variables when interpreting inflammatory biomarker changes in periodontitis.

### Strengths and Limitations of the Study

This study provides a comprehensive analysis of key inflammatory biomarkers (CRP, IL-1, IL-6, TNF-α) across all stages and grades of periodontitis, offering detailed insights into both systemic and local inflammatory responses. The significant reductions observed in these biomarkers following treatment across diverse patient groups reinforce the effectiveness of periodontal therapy in modulating inflammatory activity. Moreover, the findings are consistent with previous literature, supporting the validity and reproducibility of these inflammatory markers as potential diagnostic and monitoring tools in periodontitis.

The sample size, although adequate, may not fully capture the variability present in less prevalent stages and grades, thereby limiting the generalizability of the findings across all patient subgroups. Additionally, the absence of long-term follow-up restricts insights into the durability of biomarker changes and the sustained clinical outcomes post-treatment. Future research should prioritize longitudinal, randomized controlled trials involving larger and more diverse populations to validate these findings and to elucidate the temporal dynamics of inflammatory biomarker responses to periodontal therapy.

## 5. Conclusions

Within the limitations of this retrospective study, non-surgical periodontal therapy was associated with significant reductions in systemic inflammatory biomarkers (CRP, IL-1β, IL-6, TNF-α) across all stages and grades of periodontitis. These findings support a link between periodontal inflammation and systemic inflammatory activity. However, due to the retrospective design, lack of long-term follow-up, and incomplete control for confounders, causality cannot be established. Future prospective studies should include larger and more diverse cohorts, standardized follow-up intervals, and comprehensive behavioral and metabolic data to validate the clinical utility of systemic cytokines, particularly IL-6, as biomarkers of periodontal disease activity and treatment response.

## Figures and Tables

**Table 1:** Baseline characteristics of the study participants.

	Total with Periodontitis		Control Group		*p*-Value
Total	75	100.0	35	100.0	
Gender					
F	45	60.0	18	51.4	*p* = 0.522
M	30	40.0	17	48.6	
Residence					
Urban	50	66.7	26	74.3	*p* = 0.559
Rural	25	33.3	9	25.7	
Education					
Elementary	10	13.3	7	20.0	*p* = 0.537
Middle High school	65	86.7	28	80.0	
Age (year)	45.1 ± 10.7 23–71		30.4 ± 5.7 20–41		*p* < 0.0001
Stage					
I and II	21	28.0			
III	30	40.0			
IV	24	32.0			
Grade					
A	22	29.3			
B	34	45.3			
C	19	25.3			

**Table 2:** CRP levels at baseline and post-treatment by periodontitis stage and grade.

Stage	CRP (mg/L)	Baseline	Post-Treatment	
I and II	N	21	21	*p* = 0.0010
	Mean ± SD	1.9 ± 1.7	1.4 ± 1.5	
	Rank	0.0–7.0	0.0–7.0	
III	N	24	24	*p* = 0.0005
	Mean ± SD	2.1 ± 2.0	1.7 ± 2.0	
	Rank	0.0–9.1	0.0–9.1	
IV	N	30	30	*p* < 0.0001
	Mean ± SD	3.2 ± 3.4	2.6 ± 3.5	
	Rank	0.0–14.1	0.0–14.1	
Control gr.	N	35		
	Mean ± SD	0.5 ± 0.6		
	Rank	0.0–3.1		
Grade				
A	N	19	19	*p* = 0.0020
	Mean ± SD	2.0 ± 2.2	1.6 ± 2.2	
	Rank	0.0–9.1	0.0–9.1	
B	N	22	22	*p* = 0.0012
	Mean ± SD	2.2 ± 1.6	1.7 ± 1.6	
	Rank	0.0–7.0	0.0–7.0	
C	N	34	34	*p* < 0.0001
	Mean ± SD	3.0 ± 3.3	2.4 ± 3.4	
	Rank	0.0–14.1	0.0–14.1	

**Table 3:** IL-1 levels at baseline and post-treatment by periodontitis stage and grade.

Stage	IL-1 (pg/mL)	Baseline	Post-Treatment	
I and II	N	21	21	*p* = 0.0001
	Mean ± DS	5.0 ± 8.8	3.8 ± 6.3	
	Range	1.0–42.0	0.5–29.0	
III	N	30	30	*p* < 0.0001
	Mean ± DS	6.8 ± 10.0	4.6 ± 6.2	
	Range	1.0–30.2	0.2–20.4	
IV	N	24	24	*p* = 0.0001
	Mean ± DS	9.0 ± 15.1	8.1 ± 15.4	
	Range	1.0–56.2	0.2–56.2	
Control gr.	N	35		
	Mean ± DS	2.1 ± 2.2		
	Range	0.2–7.4		
Grade	IL-1 (pg/mL)	Baseline	Post-Treatment	
A	N	22	22	*p* < 0.0001
	Mean ± DS	6.2 ± 11.5	5.2 ± 11.6	
	Range	1.0–56.2	0.5–56.2	
B	N	34	34	*p* < 0.0001
	Mean ± DS	6.5 ± 9.4	4.4 ± 5.9	
	Range	1.0–30.2	0.2–56.2	
C	N	19	19	*p* = 0.0005
	Mean ± DS	9.0 ± 15.0	7.7 ± 13.9	
	Range	1.0–56.2	0.2–56.2	

**Table 4:** IL-6 levels at baseline and post-treatment by periodontitis stage and grade.

Stage	IL-6 (pg/mL)	Baseline	Post-Treatment	
I and II	N	21	21	*p* = 0.0002
	Mean ± DS	3.0 ± 4.8	2.6 ± 4.9	
	Range	0.2–21.6	0.1–21.6	
III	N	30	30	*p* < 0.0001
	Mean ± DS	3.4 ± 4.2	2.7 ± 3.7	
	Range	0.2–22.2	0.1–18.0	
IV	N	24	24	*p* = 0.0002
	Mean ± DS	4.8 ± 4.4	3.4 ± 2.6	
	Range	0.2–22.2	0.1–11.0	
Control gr.	N	35		
	Mean ± DS	1.9 ± 1.6		
	Range	0.4–0.6		
Grade	IL-6 (pg/mL)	Baseline	Post-Treatment	
A	N	22	22	*p* = 0.0001
	Mean ± DS	1.9 ± 1.7	3.2 ± 4.6	
	Range	0.2–21.6	0.1–21.6	
B	N	34	34	*p* < 0.0001
	Mean ± DS	3.0 ± 4.1	2.5 ± 3.5	
	Range	0.2–22.2	0.1–18.0	
C	N	19	19	*p* = 0.0010
	Mean ± DS	4.7 ± 5.0	3.4 ± 3.0	
	Range	0.2–22.2	0.1–11.0	

**Table 5:** TNF-α levels at baseline and post-treatment by periodontitis stage and grade.

Stage	TNF-α (pg/mL)	Baseline	Post-Treatment	
III	N	21	21	*p* = 0.0001
	Mean ± DS	71.8 ± 60.3	49.7 ± 51.3	
	Range	12.8–236.4	6.0–236.4	
I and II	N	24	24	*p* = 0.0001
	Mean ± DS	75.0 ± 60.4	58.7 ± 62.1	
	Range	27.6–321.0	15.0–321.0	
IV	N	30	30	*p* < 0.0001
	Mean ± DS	89.7 ± 89.7	60.4 ± 53.6	
	Range	12.8–300.8	6.0–180.0	
	Range	12.8–321.0	6.0–321.0	
Control gr.	N	35		
	Mean ± DS	64.6 ± 72.3		
	Range	14.0–319.0		
Grade	TNF-α (pg/mL)	Baseline	Post-Treatment	
A	N	22	22	*p* < 0.0001
	Mean ± DS	68.8 ± 50.9	47.2 ± 47.8	
	Range	12.8–236.4	6.0–236.4	
B	N	34	34	*p* < 0.0001
	Mean ± DS	85.0 ± 85.1	58.5 ± 50.7	
	Range	12.8–300.8	6.0–180.0	
C	N	19	19	*p* = 0.0005
	Mean ± DS	85.2 ± 73.7	65.0 ± 70.7	
	Range	27.6–321.0	17.0–321.0	

**Table 6:** Average values of CRP, IL-1, IL-6, and TNF at the baseline and post-treatment according to stages.

Stage	N	CRP (mg/L) Mean ± SD		IL-1 (pg/mL) Mean ± SD		IL-6 (pg/mL) Mean ± SD		TNF-α (pg/mL) Mean ± SD	
		Baseline	Post Treatment	Baseline	Post Treatment	Baseline	Post Treatment	Baseline	Post Treatment
I and II	21	1.9 ± 1.7	1.4 ± 1.5	5.0 ± 8.8	3.8 ± 6.3	3.0 ± 4.8	2.6 ± 4.9	75.0 ± 60.4	58.7 ± 62.1
III	24	2.1 ± 2.0	1.7 ± 2.0	6.8 ± 10.0	4.6 ± 6.2	3.4 ± 4.2	2.7 ± 3.7	71.8 ± 60.3	49.7 ± 51.3
IV	30	3.2 ± 3.4	2.6 ± 3.5	9.0 ± 15.1	8.1 ± 15.4	4.8 ± 4.4	3.4 ± 2.6	89.7 ± 89.7	60.4 ± 53.6
*p*-value		*p* = 0.225	*p* = 0.278	*p* = 0.166	*p* = 0.324	*p* = 0.025 I and II vs. III *p* < 0.05	*p* = 0.009 I and II vs. III *p* < 0.05	*p* = 0.619	*p* = 0.636

**Table 7:** Average values of CRP, IL-1, IL-6, and TNF at the baseline and post-treatment according to grades.

**Grade**	**N**	**CRP (mg/L)** **Mean ± SD**		**IL-1 (pg/mL)** **Mean ± SD**		**IL-6 (pg/mL)** **Mean ± SD**		**TNF-α (pg/mL)** **Mean ± SD**	
		**Baseline**	**Post Treatment**	**Baseline**	**Post Treatment**	**Baseline**	**Post Treatment**	**Baseline**	**Post Treatment**
A	19	2.0 ± 2.2	1.6 ± 2.2	6.2 ± 11.5	5.2 ± 11.6	1.9 ± 1.7	3.2 ± 4.6	68.8 ± 50.9	47.2 ± 47.8
B	22	2.2 ± 1.6	1.7 ± 1.6	6.5 ± 9.4	4.4 ± 5.9	3.0 ± 4.1	2.5 ± 3.5	85.0 ± 85.1	58.5 ± 50.7
C	34	3.0 ± 3.3	2.4 ± 3.4	9.0 ± 15.0	7.7 ± 13.9	4.7 ± 5.0	3.4 ± 3.0	85.2 ± 73.7	65.0 ± 70.7
*p*-value		*p* = 0.456	*p* = 0.592	*p* = 0.615	*p* = 0.819	*p* = 0.218	*p* = 0.179	*p* = 0.832	*p* = 0.519

**Table 8:** Mean values of CRP, IL-1, IL-6, and TNF at the baseline and post-treatment according to stages and grades.

Stage/ Grade	N	CRP (mg/L) Mean ± SD		IL-1 (pg/mL) Mean ± SD		IL-6 (pg/mL) Mean ± SD		TNF-α (pg/mL) Mean ± SD	
		Baseline	Post Treatment	Baseline	Post Treatment	Baseline	Post Treatment	Baseline	Post Treatment
1 and 2A	5	1.1 ± 1.4	0.7 ± 0.9	10.3 ± 17.8	7.1 ± 12.3	1.0 ± 0.8	0.7 ± 0.7	103.2 ± 74.8	54.2 ± 47.0
1 and 2B	11	2.5 ± 1.8	1.9 ± 1.9	3.5 ± 2.8	2.8 ± 3.0	4.7 ± 6.1	4.2 ± 6.4	69.6 ± 64.4	52.4 ± 64.6
1 and 2C	5	1.4 ± 1.5	1.0 ± 0.8	3.1 ± 2.0	2.6 ± 2.1	1.0 ± 1.1	0.9 ± 1.2	45.3 ± 10.4	39.3 ± 18.0
3A	13	2.2 ± 2.5	1.9 ± 2.5	9.1 ± 15.1	8.6 ± 15.3	6.1 ± 5.3	4.5 ± 3.0	80.9 ± 76.4	72.8 ± 80.4
3B	11	1.9 ± 1.4	1.5 ± 1.3	8.9 ± 16.0	7.5 ± 16.2	3.1 ± 2.2	2.2 ± 1.4	68.0 ± 36.0	42.0 ± 23.8
4A	1	3.5 ± 0.0	2.2 ± 0.0	1.2 ± 0.0	0.3 ± 0.0	3.4 ± 0.0	2.2 ± 0.0	27.6 ± 0.0	18.0 ± 0.0
4C	29	3.2 ± 3.5	2.7 ± 3.6	7.0 ± 10.1	4.7 ± 6.3	3.3 ± 4.3	2.7 ± 3.7	91.8 ± 90.5	61.8 ± 54.0
		*p* = 0.308	*p* = 0.431	*p* = 0.544	*p* = 0.620	*p* = 0.021 1 and 2C vs. 3A *p* < 0.05	*p* = 0.017 1 and 2 A vs. 3A *p* < 0.05	*p* = 0.540	*p* = 0.682

**Table 9:** Correlation between clinical and biochemical parameters.

	CRP-hs (mg/L)	IL-1 (pg/mL)	IL-6 (pg/mL)	TNF-α (pg/mL)
Bleeding on probing (BOP)	r = 0.176	r = 0.129	r = 0.074	r = 0.147
	R^2^ = 0.031	R^2^ = 0.017	R^2^ = 0.005	R^2^ = 0.022
	*p* = 0.130	*p* = 0.270	*p* = 0.530	*p* = 0.209
Probing Pocket Depth (PPD)	r = −0.087	r = −0.133	r = −0.008	r = 0.159
	R^2^ = 0.0076	R^2^ = 0.017	R^2^ = 0.00007	R^2^ = 0.025
	*p* = 0.455	*p* = 0.254	*p* = 0.942	*p* = 0.172
Clinical Attachment Level (CAL)	r = −0.283	r = −0.322	r = −0.435	r = −0.255
	R^2^ = 0.079	R^2^ = 0.104	R^2^ = 0.189	R^2^ = 0.065
	*p* = 0.014	*p* = 0.004	*p* = 0.000	*p* = 0.027

## Data Availability

The raw data supporting the conclusions of this article will be made available by the authors on request.
